# Experiences of patients with advanced chronic diseases and their associates with a structured palliative care nurse visit followed by an interprofessional case conference in primary care – a deductive-inductive content analysis based on qualitative interviews (KOPAL-Study)

**DOI:** 10.1186/s12875-024-02572-5

**Published:** 2024-09-04

**Authors:** Nadine Janis Pohontsch, Jan Weber, Stephanie Stiel, Franziska Schade, Friedemann Nauck, Janina Timm, Martin Scherer, Gabriella Marx

**Affiliations:** 1 Department of General Practice and Primary Care, University Medical Center Hamburg-Eppendorf, Martinistr. 52, 20246 Hamburg, Germany; 2 Institute for General Practice and Palliative Care, Hannover Medical School, Hannover, Germany; 3 Department of Palliative Medicine, University Medical Center Goettingen, Goettingen, Germany

**Keywords:** Palliative care, Heart failure, Dementia, COPD, Primary care

## Abstract

**Background:**

Chronic, non-malignant diseases (CNMD) like chronic obstructive pulmonary disease (COPD), congestive heart failure (CHF) and dementia in advanced stages are very burdensome for patients. Timely palliative care with strong collaboration between general practitioners (GPs) and specialist palliative home care (SPHC) teams can reduce symptom burden, hospitalization rates, hospitalization costs and overall healthcare costs. The KOPAL-study on strengthening interprofessional collaboration for patients with palliative care needs tested the effect of an intervention comprising of a SPHC nurse assessment and an interprofessional case conference. This qualitative evaluative study explores patients’, proxies’ and their associates’ motivation to participate in the KOPAL-study and views on the (benefits of the) intervention.

**Methods:**

We interviewed 13 male and 10 female patients as well as 14 proxies of patients with dementia and six associates of study participants using a semi-structured interview guide. All interviews were digitally recorded, transcribed verbatim and analysed with deductive-inductive qualitative content analysis.

**Results:**

Motivation for participation was driven by curiosity, the aim to please the GP or to support research, respectively to help other patients. Few interviewees pointed out to have expected positive effects for themselves. The nurse visit was evaluated very positively. Positive changes concerning health care or quality of life were reported sparsely. Most study participants did not prepare for the SPHC nurse assessment. They had no expectations concerning potential benefits of such an assessment, the interdisciplinary case conference and an early integration of palliative care. The majority of interviewees reported that they did not talk about the nurse visit and the interprofessional case conference with their GPs.

**Conclusion:**

Our results lead to the conclusion that SPHC nurses can serve as an advocate for the patient and thereby support the patients’ autonomy. GPs should actively discuss the results of the interdisciplinary case conference with patients and collaboratively decide on further actions. Patient participation in the interdisciplinary case conference could be another way to increase the effects of the intervention by empowering patients to not just passively receive the intervention.

**Trial registration:**

DRKS00017795 German Clinical Trials Register, 17Nov2021, version 05.

**Supplementary Information:**

The online version contains supplementary material available at 10.1186/s12875-024-02572-5.

## Background

Chronic, non-malignant diseases (CNMD) like chronic obstructive pulmonary disease (COPD), congestive heart failure (CHF) and dementia are very burdensome in advanced stages. CNMD are among the most frequent causes of death in Europe and worldwide [1–4]. Their numbers will increase in the near future. CNMD are often characterized by high medical complexity and prognostic uncertainty of their course. Timely palliative care can reduce symptom burden, hospitalization rates, hospitalization costs and overall healthcare costs [5–10]. Either way, it is provided predominantly to patients with oncological diseases (e.g., [11]), but not to patients with CNMD. Unsurprisingly, research indicates unmet palliative care needs in patients with CNMD [12, 13].

In Germany, palliative home care is divided in general (GPHC) and specialist palliative home care (SPHC). GPHC is provided by general practitioners (GPs), medical specialists and nursing services. SPHC is provided by high qualified health care professionals’ in multiprofessional SPHC teams. Intensive collaboration between GPs and SPHC teams supports the appropriate provision of care for patients with CNMD [12]. Case conferences are one option to intensify collaboration. Mitchell et al. [14] showed in a pilot study in Australia that a case conference between GPs and a SPHC team, preceded by a patient-caregiver-nurse conversation on issues of importance, can reduce the number of emergency department visits, hospital admissions and length of stay in hospitals. A strong desire for more intensive collaboration between GPs and specialist palliative care providers exists [15–17]. Either way, different barriers, e.g. insufficient communication and fragmentation [18], lack of clarity of prognosis and the hegemony of the curative approach [19], prevent a timely collaboration. Collaboration between these groups of health care providers needs to be facilitated. Well-prepared case conferences could be part of the solution [14, 20]. The (potentially positive) role of palliative care nurses or nurse practitioners in timely delivery of palliative care is widely discussed (e.g., [21, 22]). There are some indications from the field of residential aged care and palliative care that health professionals, patients and family caregivers support the idea of interdisciplinary case conferences (e.g. [14, 23, 24]). For instance, Mitchell et al. [14] report health care professionals’ enthusiasm about an interdisciplinary case conference with a preceding patient-caregiver-nurse conversation.

The KOPAL-study on strengthening interprofessional collaboration for patients with palliative care needs is a multicentre, two-arm, cluster-randomized controlled trial (RCT, [25]). It tested the effect of an intervention comprising of a SPHC nurse assessment, a brief consultation between this SPHC nurse and a SPHC physician and an interprofessional case conference of SPHC nurse, SPHC physician and GP. It aimed at reducing hospitalizations, symptom burden, medication use, and increasing patients’ quality of life and medical providers’ collaboration within 48 weeks after intervention [25]. A multiperspectival quantitative and qualitative evaluation including all participating actors was part of the KOPAL-study [26, 27]. In this paper the qualitative evaluation of the patients’ perspective will be addressed.

We aimed to answer the following research questions: What motivated patients and their proxies to take part in the study? How did the patients experience the individual components of the intervention? From the patients’ and proxies’ point of view, what has changed in patients’ health and healthcare as a result of study participation?

## Methods

We conducted qualitative interviews with patients, proxies (for persons with dementia) and their associates, and analyzed them using a deductive-inductive approach of content analysis [28] to assess feasibility and acceptance of the KOPAL-intervention.

### The KOPAL-study and intervention

The KOPAL-study [25] comprises a RCT and its quantitative and qualitative evaluation from different perspectives (GPs, SPHC nurse and physicians, and patients, their proxies and associates). Patients with COPD [29], CHF [30] and dementia [31] and dementia patients’ associates (as proxies) took part in the study. For further description of the patient population see [26]. The control arm provided patients with usual care (i.e. intervention was not offered at a later point in time) while the intervention comprises of a home-visit or (due to the COVID-19 pandemic) telephone call of a SPHC nurse, followed by a consultation between this nurse and a SPHC physician, and an interprofessional case conference (via telephone) of the GP, the SPHC nurse and the SPHC physician.

The SPHC nurses used the ´KOPAL conversation guide´ [32] to assess patients’ current life and health situation. Participants were neither instructed to prepare for the conversation with the nurse nor to discuss it with the GP afterwards. Correspondingly GPs were not instructed to explicitly discuss the case conference and its results with the patients. The SPHC nurse home visit (respectively phone call) lasted approx. 60 min. Case conferences lasted 5–60 min (on average 18 min) per patient and followed no prescribed structure. The qualitative evaluation interviews took place after the last follow-up-interview (48 weeks after baseline assessment/intervention) to avoid influencing the intervention.

### Recruitment and participants

Following a purposive sampling approach [33] we aimed at recruiting participating patients or proxies respectively and close relatives or acquainted persons of the patients (hereafter called associates). At the end of the last standardized follow up-interview all 65 study participants remaining in the intervention group (patients or proxies) were invited to participate in a qualitative interview to share their experiences with and thoughts about the KOPAL-study/intervention. Patients willing to take part in the qualitative interview were invited to name an associate to be interviewed, too. Usually these associates did not participate in any component of the KOPAL-study, but nevertheless had (some) knowledge about the patients’ participation in the study. Proxies of people with dementia were not asked to name another potential interviewee as they already fill the associate role.

Potential interviewees received verbal and written study information, a response sheet and a prepaid envelope. Interview participation was voluntary, reasons for non-participation were not collected. Persons willing to participate gave their written informed consent before the interview conduction. Interviewees did not receive an allowance.

Of those invited, 37 participants (42%) [patients (*n* = 23), proxies (*n* = 14)] were willing to take part in an interview. All 23 patients willing to participate were asked to name an associate. Seven patients did so. Subsequently seven associates were invited of which 6 (86%) participated. We interviewed 13 male patients (*n* = 8 with COPD, *n* = 5 with CHF) and 10 female patients with COPD (*n* = 2), CHF (*n* = 5) or both (*n* = 2). 14 proxies of patients with dementia (*n* = 7 wives, *n* = 3 daughters and one sister, son, husband and partner respectively) and six associates (*n* = 4 wives, *n* = 2 husbands; living together) of study participants were also interviewd. Patient interviews lasted 14–45 min (mean 26 min), interviews with proxies 18–54 min (mean 27 min) and interviews with associates 8–50 min (mean 25 min).

### Interview conduction

JW and NP conducted all the interviews using a semi-structured topic guide [34] via telephone between 02/2021 and 03/2022. Telephone interviews were favored to face-to-face-interviews due to the ongoing COVID-19-pandemic. All interviews were digitally recorded and transcribed verbatim. NP and JW were known to some, but not all study participants from the recruiting and standardized interviewing procedures of the KOPAL-study. No further relationship/dependency existed between researchers and interviewees.

### Interview guide

The interview guide was based on the research questions. The guide was available in three different versions (for patients, proxies of a person with dementia or associates). Topics were: the overall evaluation of the KOPAL-intervention, changes in health care and state triggered by the intervention, as well as the GP-patient-/-proxy/-associate-interaction following the case conference. See Table 1 for a translated version of the interview guide for patients who talked to the SPHC nurse on the phone (majority of interviewees). Corresponding guides for the interviews with the proxies and associates can be found in additional file 1 and 2.

**Table 1:** Interview guide for patients (participants)

**Introduction section**
Interviewer introduction, confidentiality, digital recording
Please give me a little introduction on yourself. How are you today? Which medical conditions bother you?
What was the motivation behind your participation in the KOPAL-study?
**Main questions**
During the KOPAL-study a SPHC nurse called to talk to you. How was your experience with that?
What changes occurred concerning your medical / health care after the phone call?
What changes occurred concerning your physical and mental state after the phone call?
What happened after the phone call between you and your GP? What have you been talking about?
How would you describe the cooperation between the SPHC nurse and your GP?
What problems occurred after the phone call of the SPHC nurse concerning your care?
What did you expect from taking part in the study?
**Closure**
Do you want to add something that we did not discuss yet?

### Data analysis

We (NP, JT and JW) analyzed the data using structuring qualitative content analysis as described by Kuckartz [28]. This kind of qualitative analysis is used to systematically extract, structure, describe and condense interviewees’ answers to open questions following designated rules. We chose a deductive-inductive approach to category-building using the same coding system for all interviews. NP, JT and JW familiarized themselves with the interview transcripts before coding. As starting point for the analysis deductive categories were derived from the interview guide and research questions by NP (in discussion with JW). Additional inductive categories were created either when existing deductive categories did not capture the content of the transcripts (e.g. new topics emerged from the material) or deductive categories needed subcategories to refine the coding system. If a relevant fragment was first identified, a category name was derived from this fragment and a description of the category was drafted, supplemented by a supporting quote. Codes could be assigned to text fragments adopting different sizes (ranging from part of a sentence to one or more paragraphs) in relation to the segment length needed to understand the content and context of the relevant accounts.

NP started the coding process with two transcripts and discussed coding with JW. JW coded two more manuscripts using and refining the coding system created by NP. When necessary additional inductive categories were created throughout the further coding process by JT, JW and NP. All changes to the coding system were discussed between NP, JW, JT and GM. Coding and category development were constantly discussed throughout the research process until the data relevant to the research questions were completely coded. To ensure intersubjective comprehensibility and credibility [35] of the analysis the results were discussed with GM on regular basis and presented and discussed at the DEGAM congress 2022. Data were managed and analysis was carried out using MAXQDA 12. Illustrating quotes in the results section were translated from German by NP and double checked by JW. “/” indicates an abrupt ending of a word or sentence.

### Researcher characteristics

Researchers’ characteristics, beliefs and assumptions can influence qualitative research and data interpretation [36]. NP: female, psychologist, experiences in qualitative research (QR) in the field of health services/care research; JT: female, student of Public Health (M.A.), experiences in QR; JW: male, public health researcher, experiences in QR, GM: female, sociologist, experiences in QR in the field of medical sociology and health services/care research.

### Ethics

The study was reviewed and approved by the local ethics committee of the Medical Association Hamburg, Germany (no. PV7090) as well as the ethics committees of the University Medical Centre Goettingen, Germany (no. 34/1/20Ü), the Hannover Medical School (no. 8815 BO K 2019), and the University of Oldenburg (no. 2019 − 145). The trial is registered in the German clinical trial register (registration number DRKS00017795; first registration 09/01/2020).

### Findings

Interviewees talked positively about study participation and the nurse visit. They described themselves as being motivated to participate by curiosity, wanting to do the GP a favor and support research to help other patients. Positive effects concerning health care or quality of life were meagerly reported. Most interviewees reported no communication about the KOPAL study or intervention with their treating and participating GPs.

All in all, 43 interviews were analyzed and coded. The results section is structured according to the research questions: (a) motivation to take part in the study, (b) evaluation of the intervention components and (c) changes in health and healthcare. Table 2 shows an overview over the main and subcategories described in the following section.

**Table 2:** Overview of categories

Main categories	Subcategories	
Motivation to take part in the study	- Curiosity - Pleasing the GP - Make a contribution to research and helping other patients - Specific care-related expectations	
Evaluation of the KOPAL intervention	- Evaluation of the SPHC nurse home-visit	- Positive evaluation - Topics discussed
	- Communication between the GP and the patient	- Communication on study participation - Communication on the palliative care nurse visit and case conference
	Changes in health and healthcare	Subjective benefit from the intervention and specific changes

### A) Motivation to take part in the study

Interviewees stated different motivations to take part in the study, most of them not related to their potential personal benefit, but to overarching goals like satisfying their own curiosity and being helpful to GPs, researchers and other patients.

### Curiosity

Participants stated that they were curious to take part in the study or flattered that the university was interested in them. Some saw a chance to learn something new or get insights into research studies by taking part in the study, like this patients’ with dementia wife (taking part as a proxy): “Y*es*,* I said I’d do it*,* why not? I mean*,* you can only learn from it.” (R1).* Nearly no interviewee seemed to be motivated by specific hopes or expectations in relation to their own health or care burden.

### Pleasing the GP

Potential participants received the invitation to take part in the study from their treating GPs. Study participants stated that they took part in the study because their GP proposed or ‘recommended’ the study to them, implying that it would be good for them to take part.

*Patient: Well participation*,* because and my GP Dr. [name] somehow recommended me*,* recommended me or I don’t know how that/*,* that’s how it came about and I didn’t want to say no*,* because I like Dr. [name] very much and he actually is a*,* I would say*,* very open-minded doctor. (R2, COPD)*

Other just participated in order to please or help their GPs (being study participants on cluster level). This impetus was mostly based on a stable positive relationship between the patients (or their proxies) and the GPs.

*Proxy: […] So because she is*,* she is very engaged and puts out her feelers in many areas*,* I will say*,* is very curious and I have already benefited from that. I just thought*,* if Ms. [name] wants to take part in such a study*,* then it is also important to her*,* and that’s why I said*,* okay*,* she looks after my mother so well*,* so we really feel in good hands there*,* […]*,* but I thought so*,* if Ms. [name] takes part there*,* then I would also like to support her there. (R3, daughter)*

In both cases it seemed as if interviewees did not want to refuse their family doctor’s request to participate.

### Make a contribution to research and helping other patients

Interviewees described that in their opinion doing research has a value in itself and should therefore be supported.

*Associate: […] well there is little stuff with regard to studies […] on COPD*,* […] from a scientific point of view that’s a relatively new disease. […] you have to be thankful and support everything there is*,* […] that there are people*,* who try to do something against it or search for or find help. Well/ And that’s why this was for us*,* well*,* if I may speak for us*,* this was self-evident*,* that we would take part. […] (R4, husband)*

Participants described themselves as motivated by the prospect of helping others, future patients or patients that are worse off than themselves.

*Patient: Well*,* I think it is very important to conduct studies*,* […] from which everybody can learn […]*,* however*,* that you are open to it for a start and help even more. That’s why I’m always willing to take part in studies. (R5, heart failure)*

Asked globally, many of the interviewed patients did not remember or state to have expected immediate specific positive effects for themselves from taking part in this research study.

### Specific care-related expectations

Many interviewees were unable to name specific expectations that motivated their study participation or stated that they had none except the abovementioned (e.g. doing the GP a favor or one’s stint for research). Some interviewees mentioned specific care-related expectations such as reduction or change of medication. For example one patient with heart failure and a long medication list stated: “*Well*,* I hoped that I would have to take less tablets. And also*,* that I would maybe a little bit less worn-out. […]” (R6).* Others expected to get better treatment in general, get recommendations with regard to physicians to consult or improvement of their health (behavior).

*Patient: Well*,* in fact*,* that what I said at the end*,* with a background of “Maybe they can help me.”*,* no? With my bronchia*,* for example*,* no? That you really say “Gosh*,* Mr. [name]*,* […]*,* maybe you could go there and there*,* to this or that physician. Or to this or that clinic. And then you let yourself get examined thoroughly.” […]*. (R7, COPD)

Another care-related expectation was to receive support and recommendations for associates or informal caregivers.

*Proxy: […] and I had expected that you would have one or the other hint*,* what to do better or how my mother’s care could get more extensive […]*. (R3, daughter)

Some interviewees reported rather unspecific expectations like getting some (unspecified) kind of help or impulses for caring for their relatives (interviewed proxies) and having another contact person. Some had misguided expectations like taking part in a trial to test a new medication.

### B) Evaluation of the KOPAL intervention

Even though recall was often limited due to the long period of time since the intervention, interviewees reported some assessments of the intervention components. The overall evaluation of the KOPAL-intervention was positive. Little communication between patients and professionals was reported.

#### Evaluation of the SPHC nurse home-visit

##### Positive evaluation

The guided conversation left room for questions not only to be asked by the nurse, but also by the interviewees. Sometimes welcome advice was given by the SPHC nurse, although that was not intended. Interviewees reported that there was enough time to discuss all topics of importance.

*Proxy: Well*,* I thought it to be very good and intimate*,* no? To some extend she counseled us on what you could do and it was very intimate somehow. In contrary to being pegged*,* queried and then we will see what we can do*,* no? […] She had also taken a lot of time to do it. Not working under time pressure. (R8, life partner)*

Interviewees described the palliative care nurse as friendly, attentive and competent. All in all, interviewees evaluated the SPHC nurse home-visit positively. Some interviewees stated that they had not benefited from the nurse visit, but they did not evaluate that negatively.

##### Topics discussed

According to the interviewees the content of the conversations with the SPHC nurse covered a wide spectrum from more general discussions of the overall situation to counselling on targeted support measures.

*Patient: Yes*,* it was a lot about my degree of care and […] how I can get other support besides my husband*,* who is my carer*,* can get other support. And I found that very good. (R5, heart failure)*

Other topics were dying and palliative care, provision of medical aids, pain and nursing services.

*Patient: Yes*,* […] that one then also possibly has such*,* an example*,* if one now has it [COPD/CHF] really extremely […] that one can then also go to such a ward*,* that one is cared for palliative*,* to die. […] Or that you might want to die at home*,* but what I have often explained in our conversations is that […] I don’t want that. That I would die here at home somehow. (R9, COPD)*

General topics included for example health, household chores, self-care or mobility. More specific topics like getting support from volunteers, application for services of the public care insurance (‘care degree’) or preparation of mandates.

### Communication between the GP and the patient

All in all the interviewees reported little communication between themselves and the GPs concerning the KOPAL study and intervention.

#### Communication on study participation

Most of the interviewees reported that there was no conversation about the KOPAL-study between them and their treating GPs. It seems, from the interviewees’ perspective, that GPs might have had too little time to discuss the subject in a consultation: *“No*,* she didn’t have time for that (laughs). […] (R5*,* heart failure).* Some GPs inquired whether the patients decided to take part in the study but never mentioned the study again as far as the interviewees remembered.

*Proxy: […] The doctor*,* Ms. [name]*,* had not commented on it at all. She had only asked at the beginning whether we had participated and whether we had received a visit from Cobra [mispronounced name of the KOPAL-study]*,* and I had said “Yes*,* that’s so and the first talks have already been held*,* also by telephone” and she agreed to that. That was enough for her. (R8, life partner)*

Other interviewees mentioned that they seldom consulted the GP during the time of the study indicating that that might have been a reason for not having discussed aspects of the KOPAL study or intervention during consultations.

#### Communication on the palliative care nurse visit and case conference

The study design did not specify whether the GPs should communicate the results of the case conference. It has been shown, that apparently only few of them have sought a conversation with their patients (or patients’ proxies). Most interviewees reported that neither the visit of the SPHC nurse nor the case conference were addressed between them and the treating GPs.

*Patient: Yes*,* I was surprised*,* but he didn’t talk to me about it*,* and I said I didn’t really have to talk to him about it. Because he has always found out everything I need in terms of my health and I have discussed it with him. But I didn’t talk it through with him. (R10, heart failure)*

In most cases neither the patients or proxies nor the GPs seem to have taken initiative to talk about the component of the KOPAL-intervention with each other.

### C) Changes in health and healthcare

Interviewees did not associate many positive effects, benefits or changes to the KOPAL-intervention. They did not mention any negative consequences due to their study participation either.

### Subjective benefit from the intervention and specific changes

Some specific changes due to the KOPAL-intervention were mentioned. The interviewees sometimes also mentioned other positive aspects or benefits from the KOPAL-study. Some patients reported changes or reduction in medications.

*Patient: […] I had announced all my tablets*,* there was a woman here with me once and she wrote down everything with my tablets*,* and the professor [incorrect reference to the SPHC physician] immediately called my doctor […]. And then he immediately said*,* “She should leave that tablet out and he’ll prescribe another one instead.“. And that really helped*,* it was very good. […] (R11, heart failure)*

Other interviewees mentioned additional household help respectively nursing service, the grant of a new care degree and associated eligibility for services by the public care insurance, more home visits of the GP, and the expansion of care to a SPHC service.

*Patient: […] since they were here and I get care allowance*,* if that’s what you mean. […] Yes*,* that’s it for the moment*,* at the moment the tablets for the whole week*,* they come on Monday from community (A) from the deaconry […] and bring them to us for the whole week*,* and we need household help […] I can’t manage that either*,* because I can’t bend down so well […] Anyway*,* now we have the money for the help we need. (R12, heart failure)*

All in all, patients, proxies and associates reported only little or often no subjective benefits that they trace back to the KOPAL-intervention throughout the interviews. “*Well*,* nothing at all in terms of medical care. Everything has remained the same.”* (R4, husband) therefore was a common statement. Most interviewees do not seem to have noticed any major changes.

## Discussion

### Main results

The KOPAL-study [25] tests the effect of a novel compact intervention (SPHC nurse home-visit and an interprofessional case conference) on reduction of hospital admissions, symptom burden, health costs, and improvement of quality of life. We evaluated the patients’, proxies’, and associates’ perception of the intervention, specifically the SPHC nurse visit, by conducting and analyzing qualitative interviews. Motivation for participation was driven by curiosity, the aim to please the GP or to support research, respectively to help other patients. Only few interviewees pointed out to have expected positive effects for themselves. The nurse visit was evaluated very positively. Positive changes concerning health care or quality of life were reported sparsely. Most interviewees reported that they did not talk about the nurse visit and the interprofessional case conference with their GPs.

### Discussion of results and comparison with existing literature

Our results show that the nurses’ home visit/phone call using the KOPAL conversation guide [32] to assess the patients’ current life and health situation and identify patients’ specific palliative care needs was very well accepted by patients, proxies and associates. Similar assessments of palliative care needs were shown to be well accepted in other studies, too, e.g. [37]. In some cases our interviewees reported to have received some advice during the structured assessment by the SPHC nurse and some patients reported changes due to having had this conversation. Therefore it can be assumed that the SPHC home visit might have a positive effect itself and can be considered a reasonable addition to conducting case conferences just as it was the case in Mitchell et al.’s pilot study [14].

We found that participants did not prepare for the conversations with the SPHC nurse. Maybe this was partly because they did not expect an individual benefit for themselves (or the associated patient) from the intervention, but were motivated by positive attitudes towards research and altruistic motives to help other as it was shown by Carandang et al. [38] for elderly patients. Interviewees reported a rather passive role in the intervention. This might also have been the case due to participants’ confusion over the role of the SPHC nurse visit and the role of the interdisciplinary case conference for their own or the associated patients’ future care. Not all interviewees seemed to have made the connection between the SPHC nurse visit and the interdisciplinary case conference. This confusion is already known from other studies (e.g., [23]). Other studies show that being asked to identify questions before such an exchange with professionals might be beneficial and that it is important explicitly explain the purpose of the intervention to increase acceptability and benefit for patients [39].

Interviewees reported lack of communication between patients and GPs about the results of the interdisciplinary case conferences. This could have hindered resolved changes in care to be implemented. Integrating a mandatory discussion of case conference results and refinement of the plan between the GP and the patient or proxy (as it was the case in Mitchell et al.’s study [14]) could have helped to realize potential changes/actions decided on in the case conference. This could increase the impact of the intervention.

### Strengths and limitations

We interviewed patients in an advanced stage of their disease, proxies of patients with dementia, and patients’ associates. We were able to obtain a comprehensive view on and a subjective evaluation of the KOPAL-intervention. Due to the randomized controlled design of the KOPAL-study we decided to schedule qualitative interviews after the last quantitative follow-up interview to prevent contamination of intervention effects and to maintain the comparability with the control group. Therefore no qualitative interview data is available from participants deceased during the follow-up period. Furthermore, the rather long time period between study enrollment, intervention and evaluation interview might have induced a reduced memory of the SPHC nurse home-visit/phone call.

## Conclusion

The results give some indication what to consider in a future implementation of the KOPAL-intervention in regular care. Most study participants did not prepare for the SPHC nurse assessment and had no expectations concerning the potential benefits of such an assessment, the interdisciplinary case conference and an early integration of palliative care. This points to the conclusion that the SPHC nurse can act as an advocate for the patient and thereby support the patients’ (and their proxies’/caregivers’) autonomy. Another way to increase the effects of the intervention could be letting the patients (and/or their proxies/caregivers) take part in the interdisciplinary case conference. This could empower patients (and/or their proxies/caregivers) to not just passively receive the intervention but to actively take part in deciding on further actions. Besides this measure to activate the patients (and/or their proxies/caregivers), GPs should actively discuss the results of the interdisciplinary case conference with the patients and collaboratively decide on further actions to be realized. Further research is needed to evaluate the effects of the proposed changes.

## Electronic supplementary material

Below is the link to the electronic supplementary material.

Supplementary Material 1

Supplementary Material 2

## Data Availability

No data are available. The data generated and analyzed during the current study are not publicly available due to the study’s assurances to participants that the full raw interview data would not be shared publicly, and that all attempts would be made to maintain confidentiality.

## Abbreviations

CHF: Congestive heart failure
CNMD: Chronic, non-malignant diseases
COPD: Chronic, obstructive pulmonary disease
GP: General practitioner
SPHC: Specialist palliative home care
KOPAL-study: Strengthening interprofessional collaboration for patients with palliative care needs – development and evaluation of a new concept
