# Supplementary material for: Experiences of patients with advanced chronic diseases and their associates with a structured palliative care nurse visit followed by an interprofessional case conference in primary care – a deductive-inductive content analysis based on qualitative interviews (KOPAL-Study)
Source: BMC Prim Care. 2024 Sep 4;25:323. doi: 10.1186/s12875-024-02572-5 (PMC11373434; doi:10.1186/s12875-024-02572-5)
Supplement: Supplementary file 1 — Supplementary Material 1 [file 12875_2024_2572_MOESM1_ESM.docx]

**Interview guide for relatives /associates (of patients without dementia)**

| Introduction section |
| --- |
| Interviewer introduction, confidentiality, digital recording |
| Please give me a little introduction on yourself. What do you do for a living? Describe your relationship to your relative / associate, who participated in our study. |
| Maybe you have talked with your relative /associate about his/her potential participation in the KOPAL-study as he /she got the invitation. What were your thoughts concerning the participation? |
| Main questions |
| As part of the KOPAL-study your relative / associate got called by a specialized palliative care nurse. How was your experience with that? |
| What changes occurred for you personally concerning the medical / health care of your relative/associate after said phone call? |
| From your point of view, what changes occurred concerning the physical and mental state of your relative/associate after said phone call? |
| What changes occurred for you personally after said phone call? What changes occurred between you and your relative / associate after said phone call? |
| What have you or your relative / associate talked about with the GP after said phone call? |
| How would you describe the cooperation between the specialized palliative care nurse and the GP? |
| From your point of view or for you personally what problems occurred due to the intervention (phone call, case conference)? |
| What did you expect for yourself from your relatives /associates participation in the study? |
| Closure |
| Do you want to add something that we did not discuss yet? |
