# Supplementary material for: Experiences of patients with advanced chronic diseases and their associates with a structured palliative care nurse visit followed by an interprofessional case conference in primary care – a deductive-inductive content analysis based on qualitative interviews (KOPAL-Study)
Source: BMC Prim Care. 2024 Sep 4;25:323. doi: 10.1186/s12875-024-02572-5 (PMC11373434; doi:10.1186/s12875-024-02572-5)
Supplement: Supplementary file 2 — Supplementary Material 2 [file 12875_2024_2572_MOESM2_ESM.docx]

**Interview guide for proxies (of patients with dementia)**

| Introduction section |
| --- |
| Interviewer introduction, confidentiality, digital recording |
| Please give me a little introduction on yourself. What do you do for a living? Describe your relationship to your relative / associate, who participated in our study. |
| What was the motivation behind your participation in the KOPAL-study? |
| Main questions |
| You (and your relative / associate) got called by a specialized palliative care nurse as part of the KOPAL-study. What do you think how your relative’s / associate’s experience was with that? How was your experience with that? What have you talked about? |
| From your point of view, which changes occurred concerning the medical / health care of your relative/associate after said phone call? |
| From your point of view, what changes occurred concerning the physical and mental state of your relative/associate after said phone call? |
| What changes occurred for you personally concerning the medical / health care of your relative/associate after said phone call? |
| What problems occurred for you personally after said phone call of the specialized palliative care nurse concerning the care for your relative / associate? |
| What did you expect for your relative / associate and yourself from taking part in the study? |
| Closure |
| Do you want to add something that we did not discuss yet? |
